# Predicting progression to dementia with “comprehensive visual rating scale” and machine learning algorithms

**DOI:** 10.3389/fneur.2022.906257

**Published:** 2022-08-22

**Authors:** Chaeyoon Park, Jae-Won Jang, Gihun Joo, Yeshin Kim, Seongheon Kim, Gihwan Byeon, Sang Won Park, Payam Hosseinzadeh Kasani, Sujin Yum, Jung-Min Pyun, Young Ho Park, Jae-Sung Lim, Young Chul Youn, Hyun-Soo Choi, Chihyun Park, Hyeonseung Im, SangYun Kim

**Affiliations:** ^1^Department of Convergence Security, Kangwon National University, Chuncheon, South Korea; ^2^Department of Neurology, Kangwon National University Hospital, Kangwon National University College of Medicine, Chuncheon, South Korea; ^3^Interdisciplinary Graduate Program in Medical Bigdata Convergence, Chuncheon, South Korea; ^4^Department of Psychiatry, Kangwon National University Hospital, Kangwon National University College of Medicine, Chuncheon, South Korea; ^5^Department of Neurology, Soonchunhyang University Seoul Hospital, Soonchunhyang University College of Medicine, Seoul, South Korea; ^6^Department of Neurology, Seoul National University Bundang Hospital, Seongnam, South Korea; ^7^Department of Neurology, Seoul National University College of Medicine, Seoul, South Korea; ^8^Department of Neurology, Asan Medical Center, University of Ulsan College of Medicine, Seoul, South Korea; ^9^Department of Neurology, Chung-Ang University Hospital, Chung-Ang University College of Medicine, Seoul, South Korea; ^10^Department of Computer Science and Engineering, Kangwon National University, Chuncheon, South Korea

**Keywords:** mild cognition impairment, Alzheimer's Disease, brain MRI, machine learning, visual rating scale

## Abstract

**Background and Objective:**

Identifying biomarkers for predicting progression to dementia in patients with mild cognitive impairment (MCI) is crucial. To this end, the comprehensive visual rating scale (CVRS), which is based on magnetic resonance imaging (MRI), was developed for the assessment of structural changes in the brains of patients with MCI. This study aimed to investigate the use of the CVRS score for predicting dementia in patients with MCI over a 2-year follow-up period using various machine learning (ML) algorithms.

**Methods:**

We included 197 patients with MCI who were followed up more than once. The data used for this study were obtained from the Japanese-Alzheimer's Disease Neuroimaging Initiative study. We assessed all the patients using their CVRS scores, cortical thickness data, and clinical data to determine their progression to dementia during a follow-up period of over 2 years. ML algorithms, such as logistic regression, random forest (RF), XGBoost, and LightGBM, were applied to the combination of the dataset. Further, feature importance that contributed to the progression from MCI to dementia was analyzed to confirm the risk predictors among the various variables evaluated.

**Results:**

Of the 197 patients, 108 (54.8%) showed progression from MCI to dementia. Tree-based classifiers, such as XGBoost, LightGBM, and RF, achieved relatively high performance. In addition, the prediction models showed better performance when clinical data and CVRS score (accuracy 0.701–0.711) were used than when clinical data and cortical thickness (accuracy 0.650–0.685) were used. The features related to CVRS helped predict progression to dementia using the tree-based models compared to logistic regression.

**Conclusions:**

Tree-based ML algorithms can predict progression from MCI to dementia using baseline CVRS scores combined with clinical data.

## Introduction

Mild cognitive impairment (MCI) indicates the transitional stage between a normal cognitive state and Alzheimer's dementia (AD) (1). The annual rate of progression from MCI to dementia reported in community-based studies is ~6% (2, 3), whereas it was as high as 15% in a clinical study (4). MCI is recognized as a very important public health problem with regard to the risk of dementia. However, MCI comprises a heterogeneous group of conditions and not all of them progress to dementia (4). Therefore, it is necessary to assess the risk of progression from MCI to dementia using biomarkers to identify patients with a high risk of progression to dementia (5).

Brain magnetic resonance imaging (MRI) is commonly used to identify structural changes related to dementia. The National Institute on Aging-Alzheimer's Association has included structural atrophy on MRI scans as a neurodegenerative marker of AD (6–8). An AD-like atrophy pattern primarily observed in the hippocampus is the well-established biomarker of AD (9). However, there is growing evidence that atrophy of other parts of the brain, such as the parietal lobe, provides additional prognostic information (10, 11). Additionally, non-AD conditions, such as cerebrovascular lesions, are also common pathologic findings (12). Considering the multiple pathologies frequently observed in cases of MCI, it is necessary to identify neuroimaging markers that simultaneously reflect neurodegeneration and vascular injury (13).

A quantified comprehensive visual rating scale (CVRS) based on brain MRI has been developed to enable a complete understanding of structural cerebral changes, such as atrophy and cerebrovascular lesions (14). The CVRS integrates the preexisting visual rating scales (hippocampal atrophy, cortical atrophy, ventricular enlargement, and small vessel disease) without losing the value of the subscales (14). Compared to quantitative volumetric measures, visual rating scales are advantageous in that they can be directly applied to clinically-acquired images in less time (15). CVRS has been validated for predicting the progression from MCI to dementia in a longitudinal follow-up study using a dataset from the Alzheimer's Disease Neuroimaging Initiative (ADNI) (16). These suggested that the CVRS scores for MCI could help identify subjects who are likely to be referred for confirmatory studies that are more invasive or expensive, such as CSF analysis or positron emission tomography (PET) scanning. The CVRS scores could also be used in clinical settings without additional advanced biomarkers except for brain MRI. However, whether this scale is also effective for predicting disease progression using other datasets and/or methodologies, such as machine learning (ML), is still unclear. Thus, this study aimed to investigate the use of the CVRS for predicting the progression from MCI to dementia over a 2-year follow-up period using ML algorithms.

Several researchers have investigated the use of ML methods for predicting the progression of AD (17). To be specific, various ML algorithms, including deep learning models, have been studied extensively using different types of data. In this study, we compared the prediction performance of four representative ML algorithms, logistic regression, random forest (RF) (18), XGBoost (19), and LightGBM (20), using a structural table dataset obtained from the Japanese-Alzheimer's Disease Neuroimaging Initiative (J-ADNI) project (21, 22). We also analyzed the most important features and the usefulness of the CVRS score for predicting the progression from MCI to dementia.

## Methods

### Subjects

The data used in this study were obtained from the J-ADNI project (21, 22). This project was approved by the ethics committee of each site where the J-ADNI data were acquired from. All subjects were native Japanese speakers aged from 60 to 84 years. Data used in this study were downloaded from the J-ADNI database on 1 May 2017. We included patients with MCI who underwent a baseline MRI scan and were followed up at least once after the initial assessment. The primary objective of this study was to predict the progression from MCI to dementia during the follow-up period of up to 2 years. A total of 197 patients from the J-ADNI cohort were finally included in this study.

The diagnosis of MCI was made based on the presence of objective memory impairment that did not meet the criteria for dementia. All the subjects had a Mini Mental State Examination (MMSE) score of 24 or higher, a global Clinical Dementia Rating (CDR) score of 0.5, a CDR memory score of 0.5 or higher, and a score indicating impairment in the delayed recall of Story IIA of the Wechsler Memory Scale-Revised (≥16 years of education: ≤ 8; 10–15 years of education: ≤ 4; 0–9 years of education: ≤ 2) (23). The diagnosis of dementia during the follow-up year was made based on the presence of memory complaints, a CDR score ≥0.5, and significant impairments in objective cognitive measures and activities of daily living. The individuals with AD met the National Institute of Neurological and Communicative Disorders and Stroke-Alzheimer's Disease and Related Disorders Association criteria for probable AD (24). At baseline, the following cognitive and functional measures based on the National Alzheimer's Coordinating Center Uniform Data Set, as used in ADNI, were examined: Digit Span, Category Fluency, Trail Making A and B, Digit Symbol Substitution Test of the Wechsler Adult Intelligence Scale III, Boston Naming Test, Clock Drawing Test, Neuropsychiatric Inventory-Q, AD Assessment Scale-Cognitive Subscale (ADAS-Cog), and Functional Assessment Questionnaire (FAQ). The participants with MCI were evaluated every 6 or 12 months. Then, clinical progression from MCI to dementia was diagnosed by a clinical site investigator at each follow-up visit and verified by an adjudication committee (25).

### Acquisition of magnetic resonance images

All subjects underwent MRI, which was performed using a 1.5-T MRI scanner. Data were collected at multiple ADNI sites as per a standardized MRI protocol, which was developed by comparing and evaluating 3D T1-weighted sequences for morphometric analyses (26). MRI acquisition and processing were performed per the standard protocol. Preprocessed T1-weighted MPRAGE MR images, a fluid-attenuated inversion recovery image, and a T2 star weighted image were downloaded from the J-ADNI database.

### Comprehensive visual rating scale

The CVRS includes scales of hippocampal atrophy, cortical atrophy, ventricular enlargement (subcortical atrophy), and small vessel disease, which summarize degenerative or vascular injury in the aged brain (Table 1). The details of each scale are described elsewhere (14) and in Supplementary file 1. These existing scales were combined in the CVRS to quantify the effects of multiple brain deficits, thus yielding a scale with scores ranging from 0 to 30 (a higher score represents more deficits).

**Table 1:** Construction of a comprehensive visual rating scale (CVRS).

	**Adopted or modified scales**	**Scale range**
Hippocampal atrophy	• Scheltens' scale for coronal image [20] • Kim and Jung's scale for Axial scale [23]	0–8 (bilaterally)
Cortical atrophy	• Victoroff's scale for frontal and temporal lobe [24] • Koedam's scale for parietal lobe [25]	0–9
Subcortical atrophy	• Donovan's scale for anterior and posterior horn of lateral ventricle [26]	0–6
Small vessel disease	• Modified Fazekas and Scheltens' scale for white matter hyperintensity [27]	0–3
	• Lacunes and microbleeds: The total number was graded	0–4

The visual rating was performed by three raters (Jae-Won Jang, Seongheon Kim, and Yeshin Kim), who were blind to the demographic and clinical information of the subjects. Each rater used a template-based scoring method (Supplementary file 2). The inter-rater and intra-rater reliability with 20 randomly selected MRI scans were 0.943 and 0.931, respectively (Supplementary file 3). Cross-sectional validation of a clinical group, including individuals with normal cognition, MCI, and dementia, was performed in a previous study (14).

### Neuropsychological data

Longitudinal neuropsychological markers, such as the MMSE score, Alzheimer's Disease Scale-Cognitive Subscale (ADAS-Cog) (27) score, and Clinical Dementia Rating-Sum of Boxes (CDR-SOB) score, were evaluated at baseline and 1-year intervals for up to 2 years.

### Statistical analysis

The independent *t*-test and chi-square test were used to examine the between-group differences in continuous variables and categorical variables, respectively. The Mann-Whitney U test was used to analyze continuous variables that were not normally distributed. Statistical significance was set at *p* < 0.05. Statistical analyses were performed using R (Version 4.1.0, The R Foundation for Statistical Computing, 64-bit platform).

### Data preprocessing

The dataset consisted of the initial diagnoses of 200 patients and those made at 6, 12, and 24 months after baseline. Our goal in this study was to predict the progression from MCI to dementia within a 24 month follow-up period. To this end, we used several clinically important features, such as demographic data, neuropsychological test results, genetic data, CVRS score, and cortical thickness, obtained during the baseline examination (Table 2) and the diagnosis made at 24 months as the target value (y label). Of the 200 patients assessed, only 197 were finally included for the analysis. A total of three patients were excluded because they did not have a diagnosis at 24 months. To examine the usefulness of the CVRS score compared to cortical thickness, the features selected from the screening data were widely used conventional variables, such as age, sex, duration of education, APOE4 genotype, and the results of cognitive function tests (CDR-SOB, ADAS-Cog11, MMSE). MRI visual rating scales, such as the total CVRS score and the hippocampal atrophy, cortical atrophy, subcortical atrophy, and small vessel disease scale scores (14) were used for the analysis. Cortical thickness was adopted as the AD signature (28), that is, the average of eight cortical thickness values computed using the MRI FreeSurfer (https://surfer.nmr.mgh.harvard.edu/). We used three datasets that consisted of clinical data, clinical data with CVRS score, and clinical data with cortical thickness to compare the prediction performance of each feature category.

**Table 2:** Baseline characteristics of the patients with MCI.

	**Stable group** **(*n* = 89)**	**Progressive group** **(*n* = 108)**	**Total** **(*n* = 197)**	***p*** **-value**
Age, years (mean ± SD)	72.9 ± 5.8	73.3 ± 5.7	73.1 ± 5.8	0.586
Female, *n*	39 (43.8%)	62 (57.4%)	101 (51.3%)	0.079
Education, years	13.5 ± 2.7	12.7 ± 2.9	13.1 ± 2.9	0.056
APOE ε4 carriers, *n*	31 (35.2%)	73 (67.6%)	104 (55.6%)	<0.001
CDR-SOB	1.3 ± 0.9	1.7 ± 1.0	1.5 ± 0.9	0.003
ADAS-cog 11	9.0 ± 3.7	12.3 ± 4.2	10.8 ± 4.3	<0.001
MMSE	26.8 ± 1.9	26.1 ± 1.5	26.4 ± 1.7	0.004
FAQ	2.3 ± 2.7	4.5 ± 4.7	3.5 ± 4.1	<0.001
CVRS (total)	8.7 ± 3.2	9.3 ± 3.9	9.0 ± 3.7	0.223
Hippocampal atrophy	3.4 ± 1.6	3.9 ± 1.6	3.7 ± 1.6	0.069
Cortical atrophy	2.1 ± 1.5	2.5 ± 1.8	2.3 ± 1.7	0.158
Subcortical atrophy	1.6 ± 1.2	1.6 ± 1.2	1.6 ± 1.2	0.858
Small vessel disease	1.5 ± 1.0	1.3 ± 1.2	1.4 ± 1.1	0.343
AD signature	2.8 ± 0.2	2.6 ± 0.2	2.7 ± 0.2	<0.001

Values are presented as mean ± standard deviation or number (%) unless otherwise stated. SD, Standard deviation; CDR-SOB, Clinical dementia rating-sum of boxes; ADAS-Cog, Alzheimer's Disease assessment scale-cognitive subscale; MMSE, Mini mental state examination; FAQ, function in daily living; CVRS, Comprehensive visual rating scale.

### Machine learning methods

To build a prediction model, we used four representative ML algorithms, namely logistic regression, RF (18), XGBoost (19), and LightGBM (20). Since the size of the dataset was relatively small, we used the leave-one-out cross-validation (LOOCV) method (29) for the analysis of the 197 patients. In addition, we used the KNN imputation method (30) to handle the missing values of one patient who did not have the APOE4 genetic test results and eight patients without the AD signature.

#### Leave-one-out cross-validation

**Figure 1:**
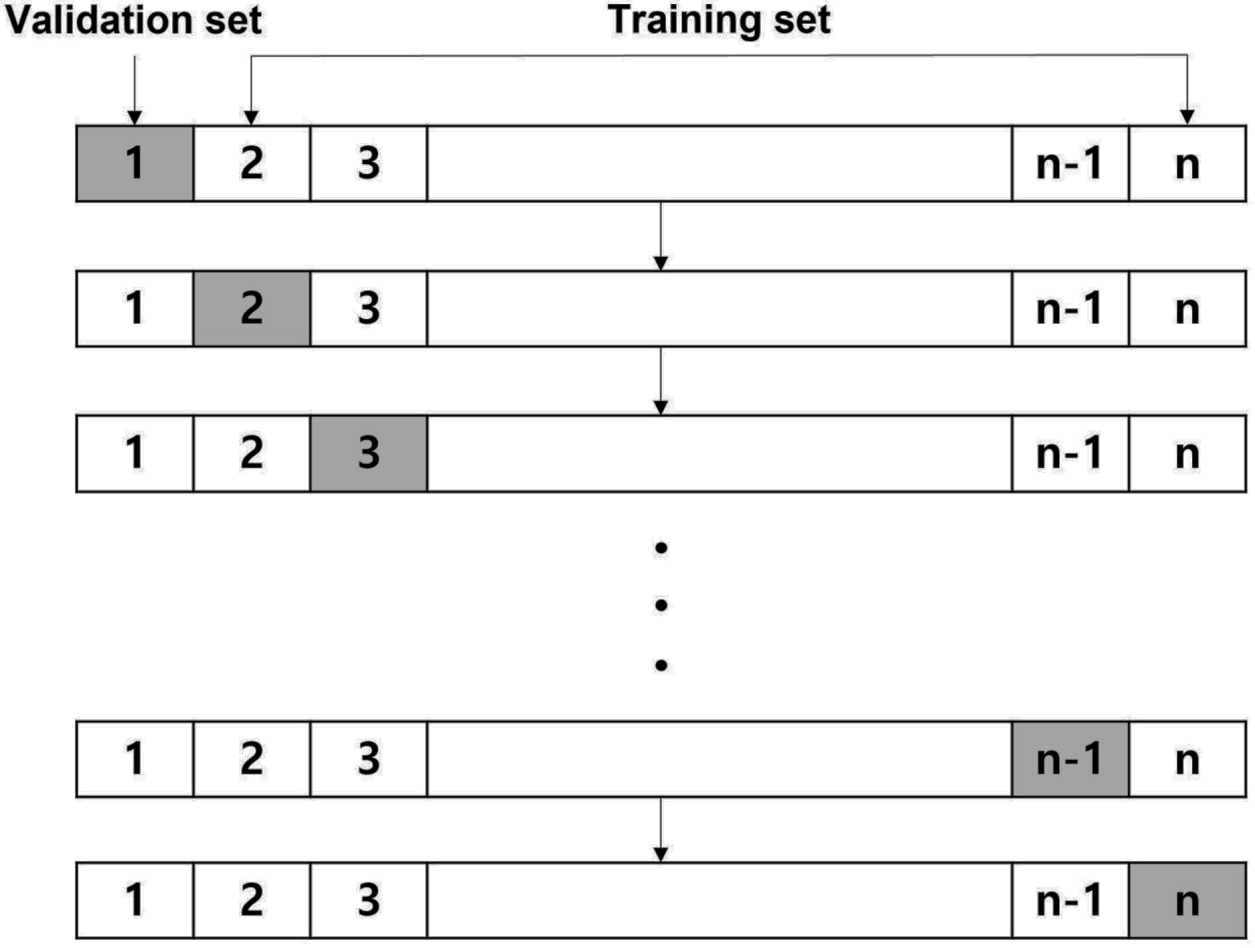
Leave-one-out cross-validation.

Leave-one-out cross-validation is a method of learning in which one data is used as a validation set and the remaining *n-*1 data as a training set. The test is performed once for all sample data (Figure 1). After a model is trained and tested a total of *n* times, the average of all mean squared errors is calculated. The LOOCV is time-consuming; however, it shows stable performance even when the size of the dataset is small. Thus, we adopted this method for our analysis.

#### Logistic regression

Logistic regression is a supervised learning algorithm that predicts and classifies a sample to a group with a probability value value between 0 and 1. It learns the relationship between the independent variables *x*_1_, *x*_2_, …, *x*_*n*_ and the dependent variable *y* as a specific function, namely *y* = σ(*w*_1_*x*_1_+…+*w*_*n*_*x*_*n*_), where *w*_1_, …, *w*_*n*_ are trainable parameters and σ is the sigmoid function, such that σ(*t*) = 1/(1+*e*^−*t*^). In linear regression, the predicted value of the dependent variable falls within the range [–∞, ∞]. In logistic regression, binary classification becomes possible by applying the sigmoid function, which always returns a probability in the range of [0, 1].

#### Random forest

An RF (18) is a machine learning method widely used to analyze structural tabular data. It is an ensemble model based on a bagging (bootstrap aggregating) method that builds multiple decision trees by using a subset of the training set. Although a single decision tree can often be overfitted, RF can prevent overfitting by using the average prediction of all the decision trees.

#### Gradient boosting methods

Gradient boosting is a kind of ensemble method that creates a strong classifier by combining weak classifiers. In this study, we used XGBoost (19) and LightGBM (20), which are widely used for analyzing structural tabular data. XGBoost is an ensemble algorithm that combines multiple decision trees and uses classification and regression trees to create them. It expands decision trees horizontally (i.e., level-wise) to reduce their depth. In contrast, LightGBM is a boosting-based ensemble algorithm that expands a decision tree vertically (i.e., leaf-wise) while continuously dividing the leaf node with the maximum loss value without balancing the tree. Since both methods have relative strengths and weaknesses, we compared their performances in predicting the progression from MCI to dementia.

#### Feature importance

For each ML model, we report their feature importance. Standard Python implementations of random forest, XGBoost, and LightGBM automatically compute feature importance while a prediction model is built. These tree-based models usually calculate the importance of each feature using the Gini impurity of each tree node (Other impurities such as entropy may also be used instead). For example, a decision tree is created so that the impurity is lowered while feature importance is maximized. The Gini impurity *G*(*T*) of a tree node *T* is calculated as follows:


$$G(T)\;=\sum_{i=1}^{n}p_{i}(i\;-\;p_{i})\;=\;1\;-\;\sum_{i=1}^{n}p_{i}^{2}$$

where *n* is the number of classes and *p*_*i*_ is the probability of each sample in *T* to belong to the corresponding class. Then, the importance *I*(*T*_*j*_) for a node *T*_*j*_ in a binary tree is calculated as follows:


$$\begin{array}{lll} I(T_{j}) & = & \;w_{j}\cdot G(T_{j})-\;w_{j\_left}\;\cdot G(T_{j\_left})\\ & - & \;w_{j\_right}\;\cdot G(T_{j\_right}) \end{array}$$

where *w*_*j*_ is the weight of node *T*_*j*_ concerning the total number of samples while *T*_*j*_*left*_ and *T*_*j*_*right*_, respectively, denote the left and right child nodes of *T*_*j*_. Finally, the importance of each feature *f*_*i*_ for a decision tree is calculated as follows:


$$I(f_{i})=\;\frac{\sum_{T_{j}\;\in \;all\;nodes\;split\;by\;f_{i}}I(T_{j})}{\sum_{T_{k}\;\in \;all\;nodes}I(T_{k})}$$

which can then be normalized as follows:


$$\begin{array}{l} I{\left(f_{i}\right)}^{norm}=\;\frac{I(f_{i})}{\sum_{f_{j}\;\in \;all\;features}I(f_{j})} \end{array}$$

The importance of a feature *f*_*i*_ on a random forest, which consists of many decision trees, is then computed as the average of *I*(*f*_*i*_)'s over all the trees. The feature importance on XGBoost and LightGBM is also calculated similarly.

### Experiments

**Figure 2:**
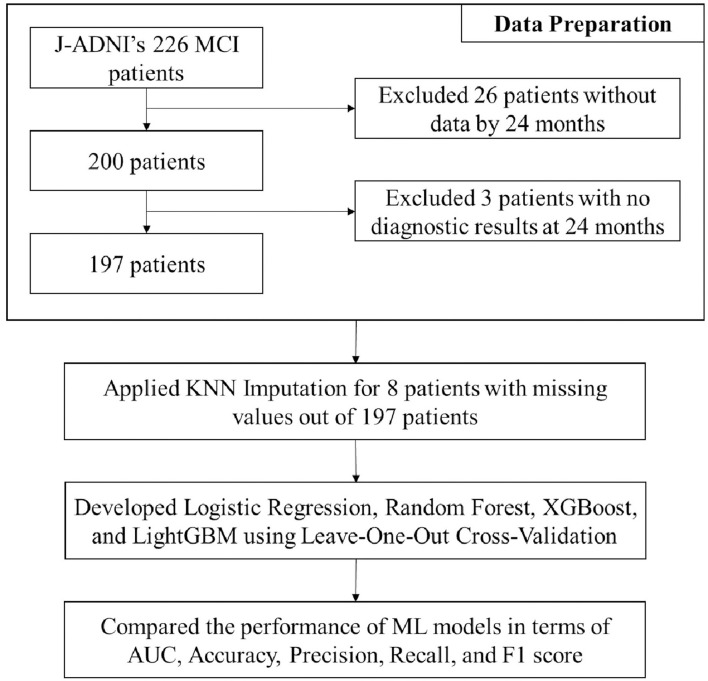
Study population and overall procedure for the experiments.

Figure 2 shows the study population and overall procedure for our experiments, i.e., from data preparation, data preprocessing, and development of machine learning algorithms to performance comparison in terms of various metrics. All the experiments were conducted on a workstation with an Intel(R) Core(TM) i7-8700 3.20 GHz CPU, 32 GB of main memory, and an NVIDIA GeForce RTX 2080 SUPER GPU. The host operating system was Windows 10 (64-bit) and all prediction models were implemented using Python 3 and the Scikit-learn machine learning library.

## Results

A total of 197 patients were included in this study. The median age of the patients was 73.11 years and 101 (51.3%) of them were females (Table 2). A total of 104 (55.6%) patients had at least one APOE ε4 allele. During the follow-up period, 108 (54.8%) patients showed progression to dementia, whereas 89 patients did not. The demographic, cognitive, and biomarker characteristics of the patients and their classification in stable MCI and progressive MCI groups based on their progression from MCI to dementia are shown in Table 2. Patients with MCI that progressed to dementia showed poorer cognitive performances at baseline, lower cortical thickness in AD signature, and were more likely to be APOE4 carriers than those that did not show progression to dementia.

### Confusion matrix

**Figure 3:**
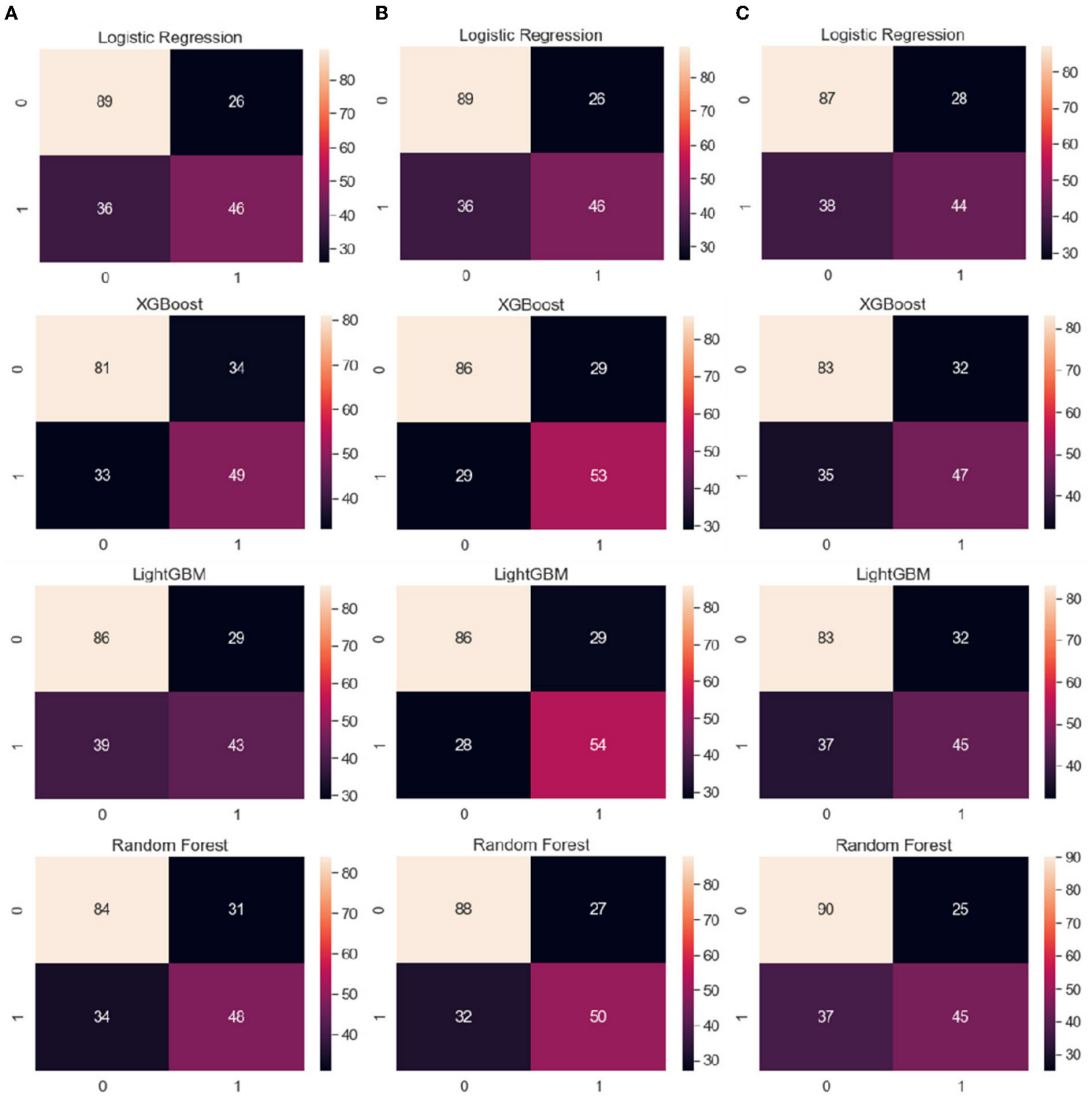
From the left, the average confusion matrix for each ML model when using the features of **(A)** clinical data, **(B)** clinical data with CVRS, and **(C)** clinical data with cortical thickness, respectively. The x-axis and y-axis represent the predicted values and the actual ground truth values, respectively.

Figure 3 shows the average confusion matrixes computed for each ML model trained using LOOCV to visualize its performance with different sets of features, namely clinical data, clinical data with CVRS score, and clinical data with cortical thickness. A confusion matrix is used to compare the actual ground truth values with the values predicted by the model, where the x-axis represents the predicted values and the y-axis represents the actual values. In the case of logistic regression, clinical data and clinical data with CVRS showed the same numbers, with the number of accurate predictions being 135 (89 + 46), which was 68% of the total data. For RF, XGBoost, and LightGBM, clinical data with CVRS showed more than 70% accuracy, which was higher than those for clinical data and clinical data with cortical thickness. For the gradient boosting models, such as XGBoost and LightGBM, more actual values were correctly predicted using clinical data with CVRS score, owing to their ability to combine different weak classifiers to create a strong classifier.

### Feature importance

**Figure 4:**
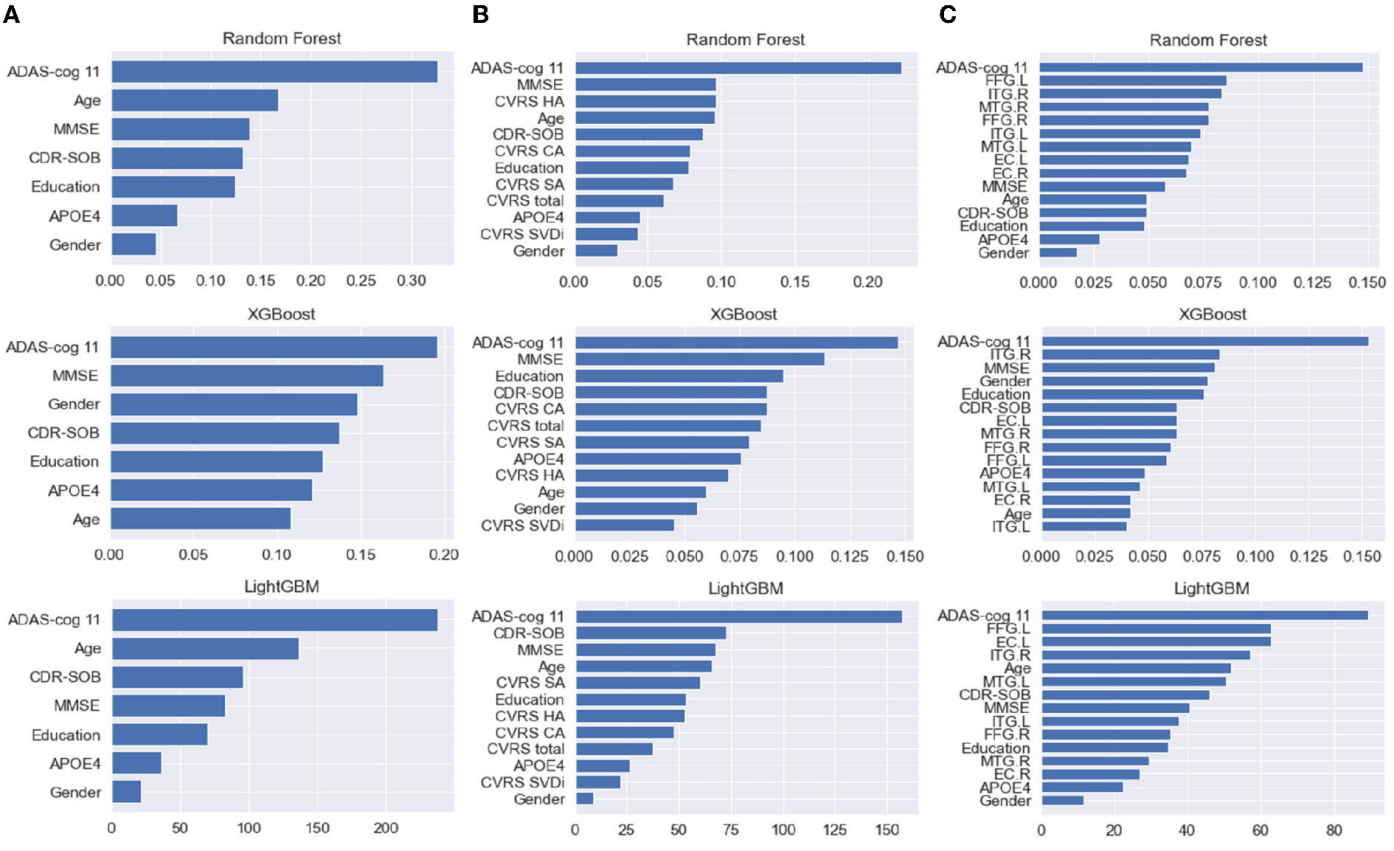
**(A–C)** From the left, the feature importance of each ML model with clinical data, clinical data with CVRS, and clinical data with cortical thickness, respectively. CVRS HA, CVRS hippocampal atrophy; CVRS CA, CVRS cortical atrophy; CVRS SA, CVRS subcortical atrophy; CVRS SVD, CVRS small vessel disease; EC.L/R, entorhinal cortex average thickness left/right; ITG.L/R, inferior temporal gyrus average thickness left/right; MTG.L/R, middle temporal gyrus average thickness left/right; FFG.L/R, fusiform gyrus average thickness left/right.

Figure 4 shows the average feature importance of each ML model, which was computed using the built-in feature importance provided by the implementation of each ML algorithm. We excluded the logistic regression model because we used it as a baseline model solely for comparing its prediction performance with those of the tree-based prediction models and thus did not eliminate multicollinearity between the input features. Regarding clinical data, ADAS-Cog 11 (cognitive function test) showed the highest importance in all models, whereas sex showed relatively little importance (Figure 4). For clinical data with CVRS score, ADAS-Cog 11 and the features related to CVRS score seemed to be helpful in predicting the progression from MCI to dementia to some extent. Regarding clinical data with cortical thickness, the indicators measured using the MRI FreeSurfer were also helpful in predicting the progression to dementia.

### Prediction results of the machine learning models

**Figure 5:**
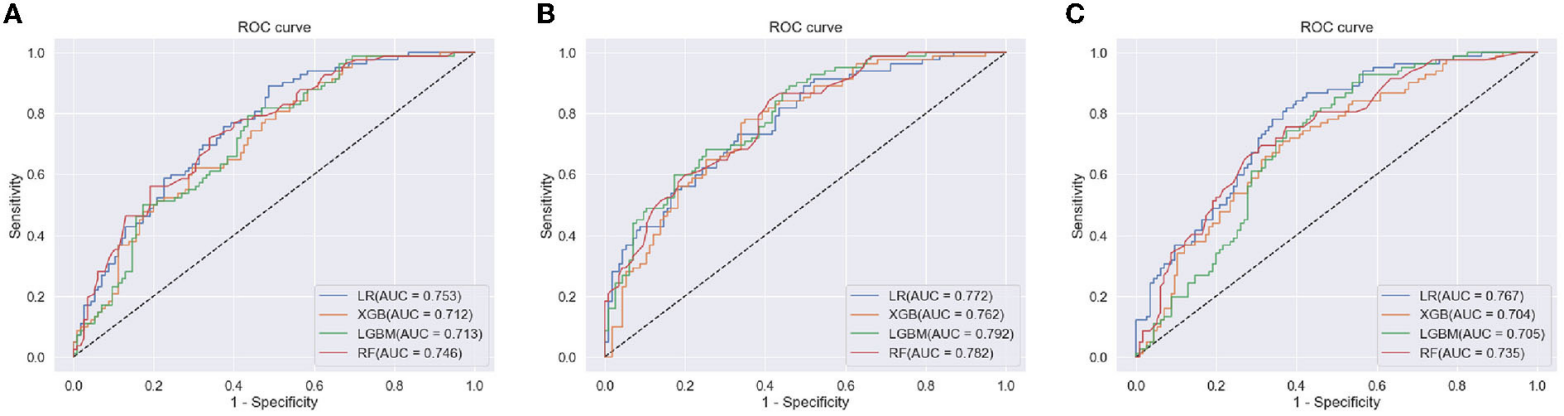
The ROC curve of each ML model for the prediction of progression to dementia within 2 years. **(A)** clinical Data, **(B)** clinical data with CVRS, and **(C)** clinical data with cortical thickness.

Figure 5 shows the receiver operating characteristic (ROC) curve for each ML model with each feature set (clinical data, clinical data with CVRS score, and clinical data with cortical thickness). Table 3 shows the comprehensive performance of each model, including details such as the area under the ROC curve (AUC), accuracy, precision, recall, and F1 score of the model. The AUC of each prediction model was the highest when clinical data with CVRS score were used, whereas the use of clinical data with cortical thickness were not quite effective as the use of clinical data only (Figure 5). For clinical data with CVRS score, which include the CVRS features, LightGBM had the highest AUC, which was 0.792, whereas, for clinical data and clinical data with cortical thickness, logistic regression had the highest AUC, which was 0.753 and 0.767, respectively (Table 3). Each prediction model achieved the highest performance in all evaluation metrics when clinical data with CVRS score were used. All tree-based models achieved a better AUC value when clinical data with cortical thickness were used than when clinical data were used, whereas logistic regression showed the opposite result. Overall, for clinical data with CVRS score, LightGBM showed the best performance in all metrics with an accuracy of 0.711, precision of 0.651, recall of 0.659, and F1 score of 0.655. In contrast, for clinical data, logistic regression showed the best performance in all metrics except for recall.

**Table 3:** The prediction performance of each ML model with leave-one-out cross-validation.

**Dataset**	**Model**	**AUC**	**Accuracy**	**Precision**	**Recall**	**F1 Score**
Clinical data	Logistic regression	**0.753** (0.686–0.820)	**0.685**	**0.639**	0.561	**0.597**
	Random forest	0.746 (0.678–0.814)	0.670	0.608	0.585	0.596
	XGBoost	0.712 (0.641–0.783)	0.660	0.590	**0.598**	0.594
	LightGBM	0.713 (0.642–0.784)	0.655	0.597	0.524	0.558
Clinical data with CVRS	Logistic regression	0.772 (0.707–0.837)	0.685	0.639	0.561	0.597
	Random forest	0.782 (0.719–0.845)	0.701	0.649	0.610	0.629
	XGBoost	0.762 (0.696–0.828)	0.706	0.646	0.646	0.646
	LightGBM	**0.792** (0.730–0.853)	**0.711**	**0.651**	**0.659**	**0.655**
Clinical data with cortical thickness	Logistic regression	**0.767** (0.702–0.832)	0.665	0.611	0.537	0.571
	Random forest	0.735 (0.665–0.805)	**0.685**	**0.643**	0.549	**0.592**
	XGBoost	0.704 (0.631–0.777)	0.660	0.595	**0.573**	0.584
	LightGBM	0.705 (0.633–0.777)	0.650	0.584	0.549	0.566

For each dataset and performance metric, we denote the highest value in bold face. For each feature set and each ML model, we show the average AUC and its confidence interval, the average accuracy, precision, recall, and F1 score. ML, machine learning; AUC, area under curve.

## Discussion

In this study, we have investigated the effects of baseline structural cerebral changes estimated using the CVRS on the progression of MCI to dementia during a 2-year follow-up period using multiple representative ML algorithms. The key finding of this study is that the ML dementia prediction models showed higher accuracy when clinical data with CVRS score were used than when clinical data alone or with cortical thickness were used. This result is in line with that of a previous study (16) on the use of visual rating scales for predicting the progression of MCI to dementia.

The CVRS scores of patients with MCI could help identify individuals who are most likely to progress to dementia without the need for additional high-cost biomarkers. The CVRS score reflects mixed pathological conditions, such as cerebral atrophy and vascular injury. Although automated image analysis of brain MRI scans has been widely used in previous research, visual rating involving scales such as the CVRS is simpler and faster, and more appropriate for individual assessment in a primary clinical setting (14, 31–33). Additionally, the CVRS is a cost-effective diagnostic tool ideally suited for implementation in clinical practice (15). In contrast, automated image analysis tools are more appropriate for detailed research that includes group analyses and a longitudinal follow-up (34). We attempted to utilize a good combination of multi-modal and highly accessible data for the predictive models by considering conventional demographic and cognitive information such as clinical data, MRI features such as CVRS score, and cortical thickness. In this study, a comparison of the predictive accuracy of the models when CVRS score was used and when the cortical thickness was used showed that CVRS had higher predictive accuracy than the cortical thickness (Table 3).

Various performance measures shown in Table 3 confirmed that for each prediction model utilized in this study, CVRS features showed more usefulness than cortical thickness features in all metrics (AUC, accuracy, precision, recall, and F1 score). Every performance measure of each prediction model was always better when clinical data and CVRS score were used together than when clinical data were used alone. In contrast, using clinical data together with cortical thickness was often worse than using clinical data alone. Regarding AUC values, the general guidelines in the book by Hosmer et al. (35) indicate that the prediction performance of all tree-based ensemble algorithms is sufficiently good when CVRS score is used, whereas it is only acceptable when cortical thickness is used. However, regarding other measures, such as accuracy, precision, recall, and F1 score, predictive performance can be improved further by considering more data or conducting hyperparameter tuning.

It is interesting to note that each ML algorithm employed different feature importance for predicting the progression from MCI to dementia (Figure 4). The results of the multivariate analysis in our previous study with US-ADNI suggested that positive amyloid PET, CDR-SOB, and CVRS are important predictors of progression from MCI to dementia (16). In this study, cognitive measures such as ADAS-Cog, MMSE, and CDR-SOB were used in all three tree-based ensemble models, with high importance for clinical data with multi-modal CVRS data. In particular, RF exploited hippocampal atrophy as the third important feature, followed by other components of CVRS. This result is mostly in line with that of a previous study in terms of the importance of the visual rating scale (15). Meanwhile, regarding clinical data with multi-modal cortical thickness data, components of cortical thickness were ranked as important features following ADAS-Cog, and most features of cortical thickness played important roles, especially for RF.

What is novel in this study compared to previous studies is that it is focused on new Asian longitudinal datasets and analytic methodologies using CVRS (Table 4). The first study used cross-sectional data from a single center that validation was performed just for test-retest reliability and clinical group differentiation (14). The following study used multisite longitudinal US-ADNI data from 63 sites in the US that showed an association between the baseline CVRS score and conversion to dementia using survival analysis (16). Finally, this study applied various ML algorithms to validate the prediction of progression to dementia using multisite longitudinal J-ADNI data from 38 sites in Japan. On top of that, we also showed higher performance of CVRS compared to cortical thickness that implicated this relatively simple tool could be used in clinical practice combined with clinical data to identify MCI subjects with a higher risk of progression. This is valuable for the clinician for the achievement of a more accurate prognosis and following a treatment plan to prevent cognitive decline.

**Table 4:** Comparison of studies using comprehensive visual rating scale (CVRS).

	**Data set**	**Study design**	**Subjects**	**Validation**
Jang et al. (14)	Data from single Korean center	Cross-Sectional analysis	NC (*n* = 65), MCI (*n* = 101), AD (*n* = 94)	• Test-retest reliability • Clinical group differentiation according to baseline CVRS
Jang et al. (16)	ADNI data from 63 sites in U.S.	Longitudinal analysis over 3 years	MCI (*n* = 340)	• Association between conversion to dementia and baseline CVRS
Current study	J-ADNI data from 38 sites in Japan	Longitudinal analysis over 2 years	MCI (*n* = 197)	• Association between conversion to dementia and baseline CVRS using various ML algorithms • Feature importance • Comparison between cortical thickness and CVRS

NC, Normal cognition; MCI, Mild cognitive impairment; ML, Machine learning.

A recent systemic review of 116 studies on the use of ML methods for predicting progression from MCI to AD showed that all the studies of MRI were conducted using automated image analysis such as cortical thickness, 3D-volumetry, tensor-based morphometry, or functional connectivity (17). Nevertheless, a balance is necessary between the advanced imaging data and ML algorithms for higher performance and the data and methods that could be available in clinical practice. Therefore, the strength of our study is further validation of the visual rating scale by adopting various ML algorithms focusing on achieving high performance using essential and easily obtainable data such as visually assessed structural MRI, demographic, and cognitive measures.

This study has some limitations. First, accuracy was relatively low compared to previously published studies. A recent systematic review showed that most studies were conducted using MRI and PET and the ADNI dataset (17). In addition, conventional algorithms, such as the support vector machine, were the most commonly used algorithms, and they had a mean accuracy of 75.4%. The highest accuracy in this study was 71.1%, which was achieved by LightGBM using demographic data and CVRS score (Table 3). The relatively low accuracy in this study may be due to the small size of the J-ADNI dataset compared to the much larger ADNI dataset. However, although the ADNI is a very useful public database that includes the data of about 1,700 subjects and has been used as a dataset in more than 3,500 publications since 2004, about 80% of the participants were Whites whereas only 2.7% of them were Asians (36). Therefore, to achieve partial generalizability of our findings for the Asian subjects, we chose the J-ADNI dataset, even though it is much smaller than the ADNI dataset. In addition, the main objective of our study was not just to achieve high accuracy using brain MRI but to compare the effectiveness of the CVRS score and that of cortical thickness for predicting progression to dementia when combined with demographic data. Second, the conversion rate (54.8% in 2 years) in this study was much higher than those reported in other studies (from 10 to 15% per year) (37, 38). A previous study speculated that this higher conversion rate of MCI in J-ADNI might happen because J-ADNI clinicians defined the clinical cutoff for AD more sensitively (25). Third, we included subjects with MCI who performed MRI at baseline without pathologic confirmation by either molecular imaging or CSF. Although J-ADNI included these data, they were not used for the analysis because these methods are either expensive or invasive. Considering the importance of cost-effective biomarker identification that is readily obtainable in a less invasive manner, CVRS of brain MRI was used for clinical implementation. Lastly, there was a decreased score of small vessel disease in the progressive group compared to the stable group although it was not statistically significant (Table 2). This was already a suggested issue that ADNI excluded subjects with a high burden of small vessel disease (16); hence, the effect of small vessel disease needs to be further validated using other datasets.

In conclusion, this study showed that for patients with MCI, a baseline CVRS score combined with clinical data are effective for predicting progression to dementia over a 2-year follow-up period. Moreover, tree-based ensemble ML models demonstrated better performances than the logistic regression model, which implies that the utility of the CVRS score can be enhanced by using appropriate ML algorithms.

## Data availability statement

Publicly available datasets were analyzed in this study. This data can be found at: Japan Science and Technology Agency (JST) National Bioscience Database Center (NBDC), http://biosciencedbc.jp/en/, JGAD000051.

## Ethics statement

The study procedures were approved by the Institutional Review Board of the Kangwon National University Hospital (No. KNUH-2017-04-012) and written informed consent was obtained from all participants or their authorized representatives.

## Japanese-Alzheimer's Disease neuroimaging initiative

Data used in this research were originally obtained by the Japanese Alzheimer's Disease Neuroimaging Initiative http://humandbs.biosciencedbc.jp/en/hum0043-v1 (led by Prof. Takeshi Iwatsubo) and are available at the website of the National Bioscience Database Center (NBDC; http://biosciencedbc.jp/en/) of the Japan Science and Technology Agency (JST).

## Author contributions

ChaP, J-WJ, HI, and SaK: designed the study. ChaP, J-WJ, GJ, HI, SP, and PK: analyzed data, composed figures, and drafted the manuscript. ChaP and J-WJ: collection of data. YK, SeK, J-MP, YP, J-SL, H-SC, SY, and ChiP: data processing. YY and HI: interpreted data for the study. HI and SaK: study supervision and critical review of the manuscript for intellectual content. All authors gave their final approval of the version to be published and agree to be accountable for all aspects of the work.

## Funding

This work was supported by the Institute for Information & communications Technology Planning & Evaluation (IITP) grant funded by the Korean Government (MSIT) (Grant No. 2022-0-01196, Regional strategic Industry convergence security core talent training business). The J-ADNI was supported by a Grant-in-Aid for Translational Research Promotion Project (Research Project for the Development of a Systematic Method for the Assessment of Alzheimer's Disease) (Grant No. 20100000001577) from the New Energy and Industrial Technology Development Organization of Japan (NEDO), by Health Labor Sciences Research Grants (Research on Dementia) (Grant Nos. H19-Dementia Research-024, H22-Dementia Research-009) from the Japanese Ministry of Health, Labor, and Welfare (MHLW), and by a Grant-in-Aid for Life Science Database Integration Project (Database Integration Coordination Program) from the Japan Science and Technology Agency (JST).

## Conflict of interest

The authors declare that the research was conducted in the absence of any commercial or financial relationships that could be construed as a potential conflict of interest.

## Publisher's note

All claims expressed in this article are solely those of the authors and do not necessarily represent those of their affiliated organizations, or those of the publisher, the editors and the reviewers. Any product that may be evaluated in this article, or claim that may be made by its manufacturer, is not guaranteed or endorsed by the publisher.
