# Supplementary material for: Predicting progression to dementia with “comprehensive visual rating scale” and machine learning algorithms
Source: Front Neurol. 2022 Aug 22;13:906257. doi: 10.3389/fneur.2022.906257 (PMC9443667; doi:10.3389/fneur.2022.906257)
Supplement: Supplementary file 1 [file Table_1.docx]

**Table S1.** Values for inter-rater and intra-rater reliability of CVRS and subscales

|  | Inter-rater (95% CI) | Intra-rater (95% CI) |
| --- | --- | --- |
| CVRS | 0.943 (0.902-0.965) | 0.931 (0.889-0.966) |
| Hippocampal atrophy | 0.861 (0.785-0.920) | 0.887 (0.803-0.938) |
| Cortical atrophy | 0.903 (0.847-0.944) | 0.940 (0.899-0.969) |
| Ventricular enlargement | 0.892 (0.831-0.938) | 0.872 (0.782-0.931) |
| Small vessel disease | 0.901 (0.846-0.944) | 0.902 (0.826-0.945) |

Values are presented as the ICC (95% CI).

*CVRS* Comprehensive Visual Rating Scale, *CI* confidence interval, *ICC* intraclass correlation coefficient
