# Supplementary figures and images for: Predicting progression to dementia with “comprehensive visual rating scale” and machine learning algorithms
Source: Front Neurol. 2022 Aug 22;13:906257. doi: 10.3389/fneur.2022.906257 (PMC9443667; doi:10.3389/fneur.2022.906257)

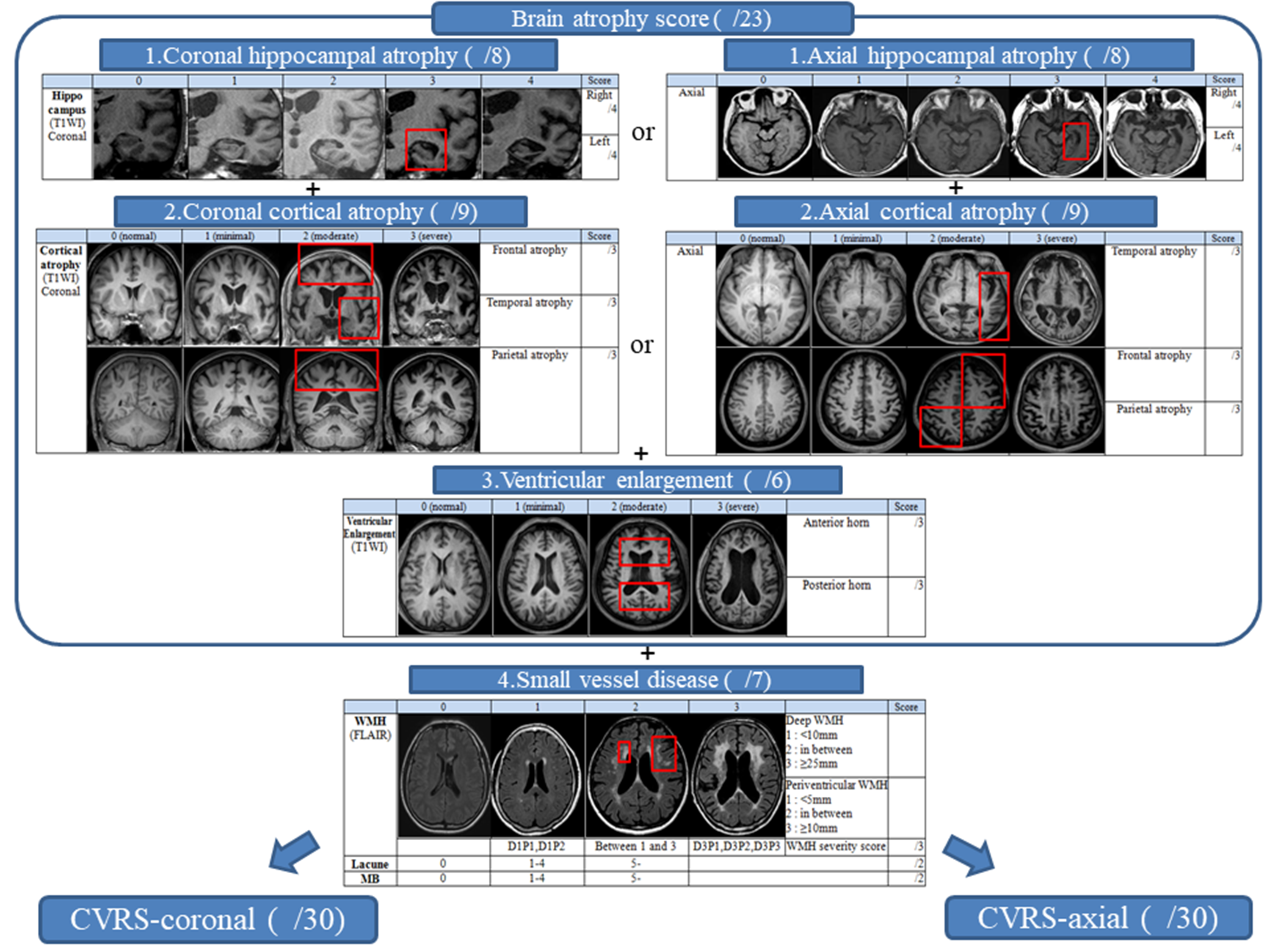

Supplement: Supplementary file 3 [file Image_1.TIF]
